# The role of physiological traits in assortment among and within fish shoals

**DOI:** 10.1098/rstb.2016.0233

**Published:** 2017-07-03

**Authors:** Shaun S. Killen, Stefano Marras, Lauren Nadler, Paolo Domenici

**Affiliations:** 1 Institute of Biodiversity, Animal Health and Comparative Medicine, University of Glasgow, Graham Kerr Building, Glasgow G12 8QQ, UK; 2 IAMC-CNR, Istituto per l'Ambiente Marino Costiero, Consiglio Nazionale delle Ricerche, Località Sa Mardini, 09170 Torregrande, Oristano, Italy; 3 Scripps Institution of Oceanography, UC San Diego, La Jolla, CA 92037, USA

**Keywords:** collective behaviour, sociality, metabolic rate, aerobic scope, ecophysiology, foraging

## Abstract

Individuals of gregarious species often group with conspecifics to which they are phenotypically similar. This among-group assortment has been studied for body size, sex and relatedness. However, the role of physiological traits has been largely overlooked. Here, we discuss mechanisms by which physiological traits—particularly those related to metabolism and locomotor performance—may result in phenotypic assortment not only among but also within animal groups. At the among-group level, varying combinations of passive assortment, active assortment, phenotypic plasticity and selective mortality may generate phenotypic differences among groups. Even within groups, however, individual variation in energy requirements, aerobic and anaerobic capacity, neurological lateralization and tolerance to environmental stressors are likely to produce differences in the spatial location of individuals or associations between group-mates with specific physiological phenotypes. Owing to the greater availability of empirical research, we focus on groups of fishes (i.e. shoals and schools). Increased knowledge of physiological mechanisms influencing among- and within-group assortment will enhance our understanding of fundamental concepts regarding optimal group size, predator avoidance, group cohesion, information transfer, life-history strategies and the evolutionary effects of group membership. In a broader perspective, predicting animal responses to environmental change will be impossible without a comprehensive understanding of the physiological basis of the formation and functioning of animal social groups.

This article is part of the themed issue ‘Physiological determinants of social behaviour in animals’.

## 1. Introduction

More than a decade ago, Krause & Ruxton [1] stated that we had little understanding of the mechanisms governing the composition and sizes of animal groups. This remains true today, despite an overall increase in research aimed at understanding collective animal behaviour [2,3]. Since this time, however, there has been a surge of interest in quantifying individual variation in physiological traits, which could provide a mechanistic perspective on our understanding of group behaviour [4–6]. The timing is right for these fields of research to experience a full conceptual convergence and empirical integration.

Group living occurs in the majority of animal taxa [1] and confers a number of costs and benefits. Some costs of group living include greater visibility to predators [7], higher aggression due to more competition for resources [1,8] and larger ectoparasite burdens [9,10]. In general, these costs are outweighed by a number of benefits including enhanced anti-predator strategies and vigilance [11,12], improved foraging efficiency [13], increased mate choice [14], reduced heat loss [15], lowered energetic cost of locomotion [16,17] and greater defence from infective stages of endoparasites [18]. Importantly, however, the balance of costs and benefits experienced by each individual within a group is context-dependent, related to the size and composition of the group and modulated by their spatial position within that group.

**Figure 1.**
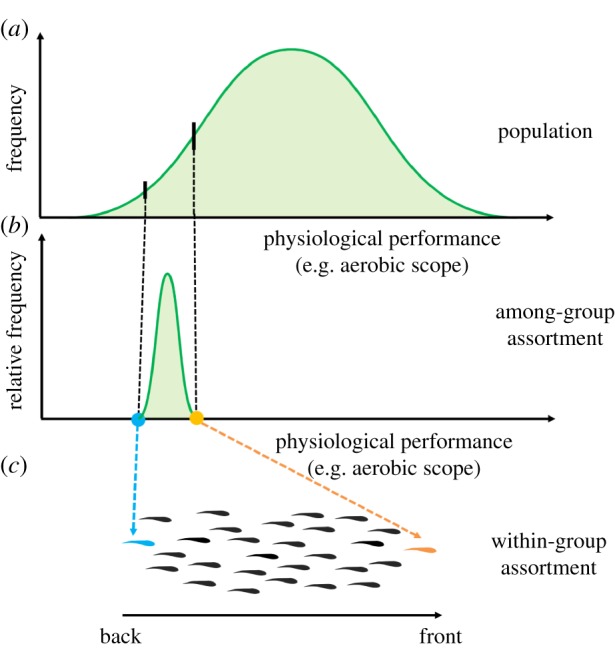
Hypothetical assortment of animal groups according to a physiological trait. (*a*) Distribution of physiological performance (e.g. aerobic scope; AS) in a population. (*b*) Among-group assortment in which social groups form within the population at various points along the continuum of the trait in question. The resultant groups have different mean levels or ranges of the trait. (*c*) Within-group assortment in which the remaining phenotypic variance within the group influences the spatial location occupied by each individual within the group. Here, fish with the higher AS are found at the front of a moving school, while fish with the lower AS are found in the back of the school, where they can benefit from hydrodynamic advantages [17].

In gregarious animal species, individuals from a population often sort into separate groups according to various phenotypic traits, such as size, sex, age and other morphological traits [1,19]. Animals within a given group will therefore often exhibit a relatively homogeneous distribution of these characteristics when compared with the population as a whole (figure 1). However, there are also wide behavioural differences among individual animals within populations, with some individuals being consistently more active, bold or exploratory across a range of contexts [6,20]. Furthermore, factors such as body size and among-individual behavioural variation are linked with a range of physiological traits [5]. There may be a direct effect of physiological traits on assortment among and within animal groups that are yet to be appreciated but that act alongside the assorting effects of body size or other morphological traits. This is especially likely given that physiology modulates locomotor performance and resource demand, both of which are fundamentally tied to the foraging and predator avoidance trade-offs associated with group membership. At present, however, the role of physiological traits in group composition and the resulting effects on social dynamics remain poorly understood.

Whole-animal metabolic traits associated with energy budgeting and physical activity may be especially relevant when considering physiological assortment of animal groups. For example, resting metabolic rate (SMR in ectotherms; basal metabolic rate in endotherms) and routine metabolic rate (RMR; SMR plus the costs of spontaneous activity) have been linked to greater food requirements and risk-taking behaviour in individuals [21,22]. Variation in SMR or RMR may influence individual social behaviour via effects on foraging requirements and hunger [23]. Maximum aerobic metabolic rate (MMR) and aerobic scope (AS, the difference between SMR and MMR) may be related to locomotor capacity and the ability to cope with environmental stressors [6,24,25]. These traits could therefore influence social behaviours by influencing the capacity for activity or escape ability. These metabolic traits may also be functionally linked. Individuals or species that perform high amounts of activity may not only have a high AS, but also an increased SMR, due to increased investment in the metabolic machinery (e.g. mitochondria, muscle mass) needed to support an active lifestyle [26]. Furthermore, although SMR can be correlated with growth rate (though the direction of this link appears to vary with context and particularly food availability [4]), AS seems to place an upper limit on food intake and growth potential [27].

Here, we describe conditions in which physiology may play a key role in the assortment of animal groups. We focus on assortment at two levels (figure 1): (i) among-group assortment, in which populations non-randomly sort into social groups based on phenotypic traits (in this case, traits related to physiology); and (ii) within-group assortment, in which phenotypic variation within a particular social group leads to differences in the spatial location of individuals or associations between group-mates with specific phenotypes. Though much of this review is theoretical, from what we know about individual variation in physiological traits, the scenarios we discuss are plausible and generate testable hypotheses regarding the potential repercussions of assortment based on physiological traits at the among- and within-group levels (figure 1). While most of our discussion can be applied to various animal taxa, fish shoals form the empirical basis for much of our reasoning. This is because they have received the most research attention with regard to both social dynamics and individual variation [1,19]. We conclude by discussing the broader ecological implications of physiological assortment of animal groups.

## 2. Physiological assortment among groups

The possibility that individuals may sort into groups according to individual physiological characteristics remains largely unexplored. Although a number of studies indicate that individuals do sort themselves into groups based on similarities in morphology and behaviour [28–30], these traits can be correlated with physiological characteristics [31–33]. Thus, similarity in appearance, body size or behaviour among individuals in a group could act as proxies for similarity in physiological traits, including metabolism, growth rate, immune function and endocrine status [26,31,32]. Because whole-animal metabolic traits are also intimately associated with individuals' energy requirements, risk of predation and locomotor capacity, they may also be directly linked to group assortment and cohesion due to the commonalities in foraging behaviour, risk and cohesive movements typically displayed by individuals in common groups. It is highly plausible that individuals might assort non-randomly to reduce conflict among group members for resource and habitat requirements, which are likely different for individuals with varying physiological needs.

There are several mechanisms that could generate physiological differences among animal groups within a population. First, individuals within a population could assort non-randomly, via either passive or active means. In passive assortment, individuals exhibit spatial or temporal overlap of similar phenotypes either due to selection of a site that suits their individual phenotype or due to commonalities in movement patterns (e.g. due to similar optimal swimming speeds or foraging behaviour). Active assortment can occur if individuals select group-mates with a similar phenotype, with groups collectively taking up residency at sites appropriate for their physiological and behavioural traits. After passive and/or active assortment occur, or even if assortment is completely random, phenotypic plasticity or selective mortality can generate or enhance phenotypic differences among groups within a population. Phenotypic plasticity of physiological traits can occur in response to environmental conditions, including an animal's physical and social environment. Selective mortality, in which certain phenotypes experience non-random mortality, shapes the phenotypic range of individuals remaining in a given habitat. These mechanisms of group differentiation have been discussed elsewhere in reference to morphological characteristics [1,19,34], but here we describe examples where these mechanisms may act on physiological traits.

### (a) Passive assortment

Links between physiological traits and habitat preferences may cause individuals with specific phenotypes to experience spatial and temporal overlap and thus coexist within the same habitat. This could result in animals becoming part of the same social group or forming more loose aggregations with relatively little social structure [1]. Commonalities in preference or tolerance for food availability and environmental conditions can cause individuals with similar phenotypes to cluster. In aquatic environments, individuals with a higher MMR or AS may be more able to occupy areas with greater flow rates, where higher aerobic swimming performance is essential to conduct normal daily processes like foraging and defence [35]. Animals with a higher SMR (and correspondingly high food requirements) or specific nutritional requirements (e.g. proportions of protein, lipid and carbohydrate) may preferentially select habitats with sufficient food availability to support these requirements and so passively associate with individuals with similar demands. As thermal tolerance limits are thought to be influenced by the ability to provide sufficient oxygen to the tissues [36], cardiorespiratory function and haematological parameters may influence the range of thermal habitats that individuals can occupy [37–39]. Hypoxic events are also becoming increasingly frequent in aquatic environments [40]. Aerobic and anaerobic capacity can affect the ability to tolerate hypoxia in fish and other aquatic organisms, and so spatial variation in oxygen availability may cause strong gradients in phenotypic variation in these traits [5]. These environmental pressures in tandem with an organism's innate physiology likely influence passive assortment of groups within specific habitats. Passive assortment could also occur as a result of intrinsic differences in movement speed or foraging behaviour among individuals within a population. This mechanism could result in patterns of assortment of physiological traits without individuals having knowledge of conspecifics' physiological requirements [41]. Variation in the amount of time spent on foraging patches, for example, because of differences in metabolic requirements, may also result in passive assortment of physiological phenotypes.

### (b) Active assortment

To maintain cohesion and synchronicity in an animal group, individuals must modify their individual behaviour and performance to match that of group members. Therefore, joining a group composed of behaviourally and physiologically similar individuals may minimize the compromises made when conforming to the locomotor activity or habitat selection of the group. For example, it would be disadvantageous for a fish to join a school consisting of individuals with a much higher or lower capacity for aerobic swimming compared with itself—faster fish could leave slower individuals behind during a predator attack or during exposure to fast current speeds, whereas slower fish may limit performance in faster individuals if group cohesion is to be maintained. It would also be beneficial to associate with conspecifics with similar tolerances to environmental stressors, as it would not be advantageous for an individual to join a group composed of animals with a tolerance for thermal extremes that exceeds its own. As a result, animals may actively choose to group with others that have similar physiological and performance traits to themselves.

A key consideration, however, is whether animals are able to evaluate the pertinent physiological traits of conspecifics via sensory cues. Subtle differences in behaviour or speed during movements could be a cue for physiological status, particularly during exposure to variation in temperature or oxygen availability. If competitive ability or motivation is in turn linked with physiological traits, then in some circumstances, there may be benefits for individuals joining groups to which they are physiologically dissimilar. It is also likely that individuals use olfactory cues for social recognition and decision-making [42,43], though the link between olfactory cues and discrimination of conspecifics based on metabolic phenotypes has not been studied. Although a gap remains in the literature on the ability of individuals to identify physiological phenotypes from sensory cues, studies indicate that individuals from social species can identify the genetic quality of conspecifics based on olfactory and visual stimuli alone, suggesting the possibility that similar signalling may exist for physiology [44,45]. Metcalfe & Thomson [46] showed that fish are able to visually evaluate competitive ability in conspecifics and choose to associate with poorer competitors. Interestingly, this example illustrates a scenario where grouping with dissimilar individuals may be advantageous.

### (c) Phenotypic plasticity

Many physiological traits exhibit plasticity in response to the prevailing environmental or social conditions. For instance, any physical environmental factor that increases the intensity and frequency of activity in animals may create a training effect that leads to improved locomotor performance [47,48]. This has been shown experimentally in laboratory studies that measured a training effect of water flow rate on aerobic metabolism and swimming performance, with higher maximum metabolic rate, gait transition speed and critical swimming speed all found [49,50]. Animals also exhibit plasticity in response to environmental stressors. Gills, for example, exhibit incredible plasticity in response to hypoxia, temperature and high sediment conditions [51–53]. These changes allow the animal to maximize oxygen uptake while limiting absorption of toxic substances. In fish and other organisms, it has also been shown that the cardiovascular system is highly plastic in response to acute challenges and can increase the capacity to deliver oxygen to tissues in response to factors such as exercise and exposure to hypoxia [54,55]. Thus, environmental conditions may create a training effect that changes individuals' physiology within a particular environment to become more similar. For example, fish living within a high-flow environment may all end up being strong swimmers, despite there being large variation in swimming ability from the outset.

Individuals within a group may also experience socially induced plasticity. Competition may cause individuals with dissimilar phenotypes to train up or down to match the group's performance, leading to intergroup differences in physiological traits. Within most species, there is consistent variation in behaviour and physiological traits [6,56,57]. Despite this variability, animal groups such as bird flocks, fish schools and insect swarms exhibit remarkable synchronous behaviour. In fish schools, for example, individuals swim at approximately the same speed and exhibit simultaneous group responses to changes in environmental factors such as hypoxia [58,59]. This suggests that school members shift their individual behavioural responses towards a collective common-ground [60]. This convergence in physiology could occur due to adjusted levels of activity and food intake to match the rates of other group members [61]. There may also be complex feedbacks which obscure the cause and effect relationship between metabolic traits and social behaviours or dominance [62]. For instance, differences in social status can alter metabolic traits due to endocrine effects and social stressors, or prolonged differences in food intake between dominant and subordinate individuals [63,64]. This could generate within-group differences in traits that did not previously exist and act to reduce physiological homogeneity within groups.

### (d) Selective mortality

Variability in physiological phenotypes could also vary due to differences in selective pressure among habitats [65]. Previous studies have illustrated differential survival among individuals with varying locomotor performance [66], and there is evidence that predation pressure may select for reduced metabolic rates in wild guppy populations [67]. However, behavioural phenotypes may not experience a uniform degree of selective pressure across habitat types. For instance, slower performing individuals may experience a higher degree of mortality, and hence be selected against, in high-flow but not low-flow regimes [68]. Traits such as growth rate, size at settlement and post-larval duration influence survival in fishes, but the strength of selection on these traits varies among sites depending on environmental conditions [69]. Selection on growth rate, swimming performance and dominance could produce correlated selection for various aspects of metabolism, endocrine function and neurophysiology in fishes [25,70]. Lastly, habitats with a high abundance of parasites may favour individuals with strong immune function and high parasite resistance, that can sustain function despite parasite infection [71].

Importantly, these four mechanisms of physiological differentiation among groups are not mutually exclusive and likely act in concert. Animals exhibit a suite of physiological traits that may be acted on by conflicting individual mechanisms. For instance, MMR may be altered by phenotypic plasticity due to a training effect, while SMR may exhibit passive assortment due to limitations from food availability. Selective mortality may act on individuals located within a specific habitat, but passive assortment may have determined which broad phenotypes preferred to associate with that habitat in the first place. In addition, individual traits may be acted upon by multiple mechanisms. Growth rate, for example, which can be tied to SMR and AS, can influence an individual's survival and selective mortality due to predation. However, active assortment based on growth rate may also occur, due to a preference to group with similarly sized individuals. In addition, there are likely unforeseen mechanisms in addition to those listed here that may impact the degree and root cause of physiological assortment within and between habitats.

## 3. Physiological assortment within groups

Despite the potential for relative homogeneity among groups, any remaining variation within the group is also likely to lead to a degree of within-group assortment and variation in spatial positioning. This form of assortment may lead to a heterogeneous spatial distribution of physiological phenotypes within animal groups. In groups with large variability in physiological phenotypes, differences in locomotor performance, environmental tolerances or nutritional requirements could result in positional (active or passive) biases, group splintering and the emergence of multiple subgroups. Here, we discuss specific mechanisms by which within-group assortment may occur, focusing on examples within teleost fishes.

### (a) Body size in relation to locomotor performance and energetics

Body size is an individual characteristic that can influence both the decision to join a group and what position to assume within the larger group. In fishes, a large body of work has illustrated individuals' preference to group (i.e. school) with similarly sized conspecifics [30,72]. Size influences physiological performance in terms of both maximum speed (e.g. in avoiding predators [73]) and cruising speed (e.g. optimal swimming speeds [74]). This variation in speed, in turn, may cause within-school sorting. In addition, spontaneous swimming speeds have been used to test the hypothesis of speed as a constraining factor on coexistence of multiple species within a single school [75]. Cruising speeds in nature are typically well below the aerobic limits of swimming speeds [76]. Therefore, small differences in size may not constrain the ability of fish of a given species to be part of the same school. However, fish of various sizes are likely to have different optimal swimming speeds (*U*_opt_, i.e. the speed at which cost of transport per unit distance is minimized [74]). Hence, if fish of different sizes all swim at the same speed (as is the case in a coordinated school), some individuals may incur an additional cost of swimming due to divergence from their own *U*_opt_. It is also possible that those with a lower *U*_opt_ sort to the back of the school as a result, to take advantage of the hydrodynamic advantages of swimming in a group [17,77]. Alternatively, variation in size within a school may be compensated by variation in performance, which would allow individuals of different sizes to school together at no additional cost as all individuals would be swimming near their *U*_opt_ [78]. This is an area that needs further investigation, especially in terms of studying within-group variation in wild schools.

### (b) Metabolism and aerobic capacity

Variation in metabolic demand could affect the spatial positioning of fish within groups. It has been observed that food-deprived fish spend more time near the front of moving schools, presumably to gain access first to encountered food items [79,80]. In an analogous manner, fish with a higher metabolic rate may prefer the front of schools, although Killen *et al*. [17] found no link between SMR and spatial position in swimming schools of grey mullet. There may also be other contexts in which metabolic rate influences the spatial positioning of individuals within groups. For example, on coral reefs, obligate coral-dwelling fish species (i.e. damselfishes and cardinalfishes) form shoals in and around coral colonies [81,82]. Within these groups, there is a trade-off between remaining close to the coral for safety and venturing away from the coral shelter to access food items in the water column [83]. Potentially, the fish on the edges of this group, that venture furthest away from the coral shelter, may exhibit a higher metabolic rate relative to their shoal-mates, but this possibility has not been examined.

**Figure 2.**
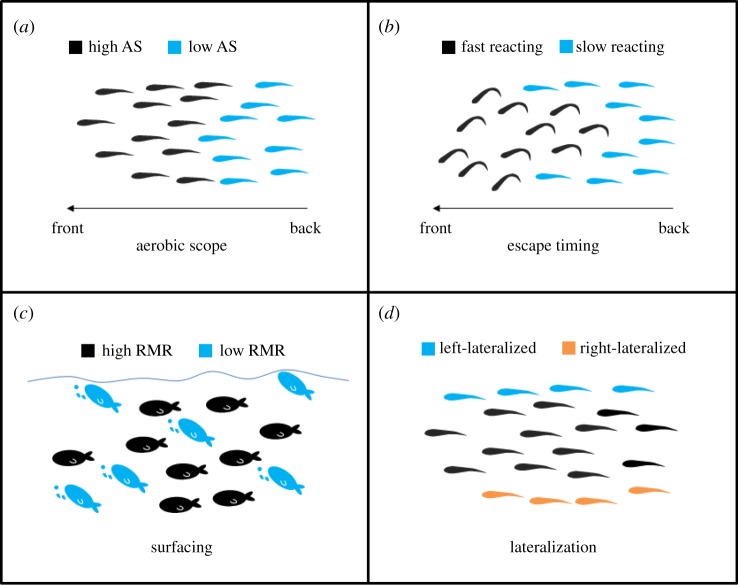
Four potential mechanisms of within-school sorting. (*a*) At fast swimming speeds, fish with low AS (blue individuals) tend to occupy positions at the back of the school, while fish with high AS (black individuals) are in the front [17]. (*b*) In small schools of golden grey mullet (*L. aurata*), fish in the back and near the edge of the school (blue individuals) tend to be the last ones to show an escape response to a threat, while fish in the front and centre (black individuals) tend to be the first to react [84]. (*c*) Fish with low RMR (blue individuals) tend to be the first ones to swim to the surface and perform ASR [85]. (*d*) Fish that are left- (blue individuals) or right-lateralized (orange individuals) occupy positions on the right or left of the school, respectively. Non-lateralized fish (black individuals) tend to stay in the centre of the school (largely based on [86]).

Aerobic capacity and swimming ability also appear to influence the spatial positioning of individuals within groups. Considerable variability in AS occurs within schools of wild caught grey mullets [17]. Interestingly, these differences were the basis for intra-school positional preferences in haphazardly sorted small schools tested in the laboratory. When swimming at relatively fast speeds, individuals with a higher AS and higher aerobic swimming capacity were leading at the front of the school and those with a lower AS were more often found located towards the back of the group [17] (figure 2). A major advantage of having a high AS may be the ability to swim at the front of the school while simultaneously feeding and diverting metabolic capacity to digestive costs (specific dynamic action [87]). It is possible, however, that fish in anterior positions may shift towards the back of schools as they become satiated. This would allow them to not only reduce predation risk but also the energetic costs of swimming, if they are able to position themselves to take advantage of the vortices shed by the group-mates ahead of themselves [16,88]. Notably, recent work has shown that individuals with a higher AS may occupy posterior positions within freely swimming schools moving at low routine speeds (A. Ward 2014 & 2015, unpublished data). It is possible that the magnitude and direction of the effect of AS on spatial locations within schools is dependent on factors such as movement speed. There may also be species-specific differences in the effect of metabolic traits on spatial positioning within shoals. Regardless of the direction of any effect of AS or swimming capacity on spatial distributions within groups, this sort of structuring could lead to a splintering of moving schools into smaller groups in situations where the main group is forced to perform aerobic swimming more quickly (e.g. during high flow rates). This is an example of a process by which within-group assortment could lead to differences in traits among groups.

Future work could investigate how links between positional preferences and metabolic traits vary with environmental context. Exposure to hypoxia should, in theory, increase within-group assortment due to constraints on MMR and thus AS—individuals with an increased MMR should be more able to occupy their preferred position within a group when compared with individuals that are more constrained. In dense schools, the biomass of fish can actually remove enough oxygen from the water such that fish towards the back of the school experience reduced oxygen availability [89]. In these cases, smaller groups may break off from the main school, so that individuals can access more oxygen. Individuals may also face a trade-off between increased oxygen availability and predation risk at the edges of moving or stationary shoals, with fish with a higher oxygen demand spending more time at the group's periphery [90]. The effects of thermal acclimation on links between physiology and within-group sorting are likely to be complex. The effects of AS on spatial positioning within groups appear to be greatest when fish are challenged by swimming at relatively high speeds. If fish are acclimated to a warmer temperature, the speed required for within-group assortment to occur might be higher if their swim performance increases with temperature, at least until the thermal optimum for AS and swim performance. It is possible, however, that individuals with an elevated SMR may have an increased motivation to move towards the front of schools at higher temperatures, to satisfy their elevated energetic demand through increased access to food. Similarly, within stationary shoals (i.e. the coral reef fish examples presented above), increased temperature could increase the need for individuals that are most sensitive to thermal increases to occupy group edges.

### (c) Escape timing

Another example of within-school sorting due to individual physiological traits is the timing of the escape response following a predator attack [84] (figure 2). Individual golden grey mullet (*Liza aurata*) in small schools (10 individuals) have been shown to escape in a non-random order, with individuals that were, for example, either first or last to react to the threatening stimulus tending to do so repeatedly in sequential stimulations [84]. Marras & Domenici [84] found that this startle order was correlated with individual positional preferences within the school, which, based on previous work [17], are likely to be physiologically driven because spatial positions in the same species are related to AS. Fish in the front and central position of the school were more likely to be the first to respond to a threat than fish in the back and near the edge of the school. As a consequence, any attack on relatively small schools of grey mullet in nature may result in sorting of school members based on their repeatable reaction order. This component of the within-school heterogeneity is likely to have important implications for schools of prey fish and the trade-offs in positions between vulnerability and foraging benefits [77]. In large schools (greater than 50 individuals), individuals near the threat tend to be the first responders and generate a wave of reaction via information transfer [91–93]. However, little is known about the potential relationship between positional preference and startle order in large schools; therefore, this is an interesting area for future work.

### (d) Surfacing

Many coastal fish species may experience recurrent hypoxia as a result of eutrophication and related disturbances [94]. Differential physiological tolerance to hypoxia and the related behavioural response, aquatic surface respiration (ASR), is a potential mechanism that can create within-school sorting (figure 2). Work by Killen *et al*. [85] has illustrated that the tendency to reach the surface during ASR in European sea bass, a schooling species, varies greatly among individuals and is related to the individual's RMR. However, ASR presents a trade-off between acquiring sufficient oxygen under hypoxic conditions and the increased exposure to aerial predation that it induces [95,96]. Therefore, individuals tend to delay ASR in the presence of predators [95,97]. Hence, differential ASR timing may lead to sorting within schools and, ultimately, differential selection in environments with greater predation pressure. Work on a number of schooling species suggests that ASR tends to be synchronized [95,98], which may minimize the ASR-induced sorting due to variation in hypoxia tolerance.

### (e) Lateralization

Another important mechanism that may result in positional sorting within a school is the individual's lateralization tendency, in which individuals preferentially turn either to the right or left during behavioural tests [86,99] (figure 2). In terms of position sorting, lateralized individuals tend to occupy central positions, while non-lateralized fish were found most often at the periphery [99]. Another study found that strongly lateralized *Malatonenia* spp*.* (two species) were found in peripheral positions with the exception of female *Malatonenia nigrans*, which displayed the reverse pattern [86]. It is hypothesized that lateralized fish swim at the periphery of the school to keep the majority of their school mates within their preferred visual field [86]. A question remains as to the degree of heterogeneity in laterality in wild schools. Early work suggests that individuals within schools may tend to exhibit a similar lateralization tendency. Although gregarious species tend to be lateralized at the population level (i.e. all individual turning in one direction), there are examples in the literature of schooling fish from wild populations that are non-lateralized at the population level [100,101]. Therefore, it is possible that the members of a school may exhibit a variety of lateralization tendencies. Bibost & Brown [86] suggest that a mix of lateralized phenotypes in a school might increase individual fitness during social interactions. For example, left- or right-lateralized individuals at the periphery will be more effective at responding to left or right stimuli than non-lateralized individuals, while lateralized individuals in the centre will be equally effective at responding to stimuli (neighbours) from either sides. The degree to which the effects of laterality on spatial preference within schools interact with factors such as feeding motivation or locomotor capacity remains unknown and is an interesting area for future work.

## 4. Consequences of physiological assortment

The costs and benefits of group membership are dynamic and vary with group composition and ambient environmental conditions [102–104]. The previously described mechanisms of among- and within-group sorting are likely to dictate which phenotypes are present within groups as well as the functioning of groups after formation. This will then have downstream effects on a number of ecological and evolutionary processes. At the individual level, there are likely trade-offs associated with any physiological assortment that might occur among and within groups with the optimal arrangement being dictated by the individual phenotype involved (e.g. high-or low-performance phenotypes) as well as the prevailing environmental conditions (table 1).

**Table 1.** Summary of potential costs and benefits of among-group assortment for individuals based on physiological traits (i.e. uniformity of a given physiological trait within fish schools).

ecological context	benefits	costs
predator avoidance/foraging	decreased oddity effect under predatory attacks increased information transfer and synchrony during coordinated escapes similar energy and nutritional requirements, thus group members spend the same amount of time foraging and searching for similar food sources	increased foraging competition among individuals with similar metabolic demand, increased aggression reduced chance to outpace group-mates when fleeing predators for high-performance phenotypes increased number of individuals required for optimal group size for low-performance phenotypes
group composition	increased cohesion in moving groups if all members have same swimming ability similar environmental tolerances and responses to stressors and so reduced exposure to non-optimal environments when conforming to group behaviour	decreased ability to occupy preferred spatial position within group; possible within group competition for spatial locations decreased niche differentiation within group (i.e. many fish may compete to be leaders in groups of high-performance individuals), possibly reducing group cohesion
resource allocation	minimize energy expenditure if all fish have similar optimal swimming speed for low-performance phenotypes, matching behaviour of group may decrease energy allocation to activity and to somatic growth and more to reproduction	higher competition for preferred position can increase shuffling rate while swimming, thus energy expenditure for high-performance phenotypes, matching behaviour of group-mates may increase energy allocation to activity and somatic growth, thus decreasing reproductive allocation

### (a) Group composition and optimal group size

**Figure 3.**
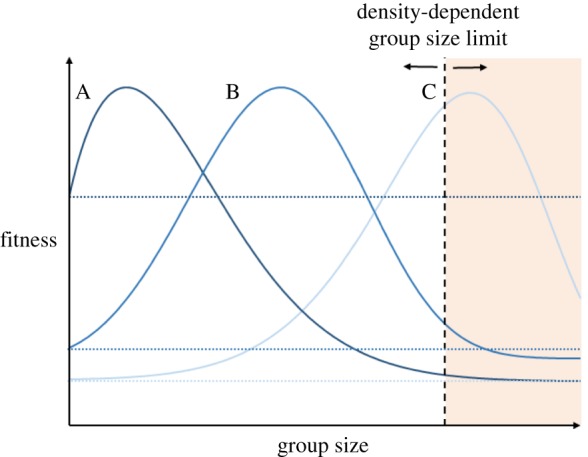
Theoretical representation of changes in fitness with group size for individuals with different energetic demands and physiological capacities for maximum levels of aerobic metabolism. Each peak represents the point at which fitness is optimized: foraging efficiency (locating foraging patches) and predator avoidance quickly increase but returns diminish as group size grows. Simultaneously, the costs of grouping increase exponentially with group size due to competition among group-mates for available food sources. Curve A represents a high-performance individual with a high maximum metabolic rate, which allows a high locomotor performance but a correspondingly high baseline metabolic rate to support this capacity. For this individual, fitness should be higher at lower group sizes due to a decreased requirement for the anti-predator benefits of grouping and an increased need to secure food. Alternatively, curve C represents a low-performance individual with a low metabolic demand. This individual should prioritize safety over foraging, due to decreased locomotor abilities and a reduced need for food. Curve B represents an intermediate individual. Dotted horizontal lines represent fitness of each individual when they are without group-mates (i.e. the *y*-intercept of each curve when group size is equal to 1). The elevation of this intercept and the curves for each phenotype will be modulated by environmental conditions. For example, under conditions of low food availability, fitness of the high-performance phenotype would theoretically go down and the fitness of the low-performance individual would go up. The dashed horizontal line represents a population-level cap on group sizes that is expected to occur due to population density. At very low population densities, low-performance phenotypes may be unable to achieve group sizes that would maximize their fitness.

The physiological phenotypes of group members may strongly affect group dynamics and modulate how the benefits gained interact with group size. For each individual that joins a group of conspecifics, their impact on the cost of food sharing increases at a faster rate than their proportional contribution to group defence (figure 3). Thus, there should be an optimal group size beyond which the proportional fitness advantages of group living decrease. However, as long as each individual incurs greater benefits than costs from group living than it would from a solitary lifestyle (see dotted horizontal lines in figure 3), individuals should still opt to join the group. A key assumption in this scenario is that all animals within the group are phenotypically similar. In reality, however, an individual's willingness to join a group should vary depending on its baseline fitness and relative competitive ability [105], which in turn may be linked to underlying physiological traits. Individuals with a high ceiling for MMR, for example, will likely have a high locomotor ability and may be more able to escape predator attacks [108]. At the same time, however, possessing the metabolic machinery to support an increased MMR can increase basal energy requirements [26,57]. For these reasons, higher performing individuals may optimize fitness in smaller groups, where there is less competition for food, at the cost of increased predation risk (figure 3). By contrast, poorer performing individuals with lower energy requirements and reduced escape abilities may prioritize a safety in numbers approach, with their fitness optimized at larger group sizes (figure 3).

Importantly, the physiological and behavioural composition of the target group may also modulate competition within groups, and therefore could dictate optimal group size [107]. The effect of body size, for example, has been studied in terms of its effects on intragroup competition and group size [106]. Many of the same arguments can be transferred to whole-animal physiological traits that might affect competitive ability or dominance, such as metabolic rate or AS [108,109]. For example, lower performing individuals should be reluctant to join groups of higher performing individuals because they are likely to be outcompeted or left behind during a predator attack if they have a limited capacity for locomotion. Given the option, therefore, they may be more likely to join a group of individuals with a similar energetic demand or performance capacity. Interestingly, however, these constraints may not apply to high-performance individuals. For them, it may be advantageous to join a group of lower quality competitors that will be poorer at securing food and be more easily targeted during predator attacks on the group [46].

It can often be disadvantageous for individuals to join groups composed of phenotypically different conspecifics because they may be singled out by predators due to the ‘oddity effect’. The oddity effect has been illustrated time and again for morphological traits such as body size [28,110–112]. Physiological traits may be more cryptic than body size, however, and so higher performance individuals within a relatively low-performance group should be less constrained by the oddity effect unless a higher performance capacity is associated with obvious behavioural differences that will draw predators' attention [85,108]. Lower performance individuals, on the other hand, should only join a group of higher performance individuals if there is some benefit for finding food patches that can be exploited that outweighs their competitive disadvantage. As a result, we can expect that some individuals will be more selective about group-mates than others, and specifically, that there may be a greater benefit to group with physiologically similar conspecifics for lower performance individuals.

The relationship between group composition and group size is likely context-dependent. For instance, individuals with a high metabolic demand may have a fitness advantage in habitats where food is abundant but will be at a disadvantage when food is scarce [4]. This will in turn lead to differences in the switch point at which it will be beneficial to join a group versus remain alone (i.e. differences in the ‘Sibly number’, as per Krause & Ruxton [1]). In addition, abiotic stressors such as thermal variation or hypoxia may amplify existing phenotypic variation within populations and potentially lead to stronger among- or within-group assortment [6]. Ultimately, the maximum possible group size will also be constrained by population density. As such, the physiological phenotypes present within groups of various sizes and the degree of homogeneity that is measured could be confounded by density-dependent life-history traits and associated effects on physiology. In addition, at lower population densities, it may not even be possible for lower performance individuals to achieve group sizes that theoretically maximize their fitness.

### (b) Leadership and group cohesion

The degree of physiological heterogeneity within groups should impact the degree of coordination and cohesion that a group displays. By definition, animals within the same social group will engage in similar activity and foraging levels and be exposed to comparable habitats and levels of risk, despite the fact that individual animals vary greatly in their behavioural tendencies (e.g. willingness to take risks) and physiological requirements for energy [5,20,57]. To function as a unit, individuals within a group must make compromises, which deviate from their own preferred behaviours and settle on a collective common-ground. Couzin *et al*. [113], for example, theoretically demonstrated that groups opt for the average preferred action when differences among individuals are small. When differences in preference are large, however, the majority preference is performed, implying that many individuals sacrifice their own preferred action to remain with the group. Groups of individuals with similar physiological traits and requirements should minimize the conflicts of interest, exhibit greater coordination and increase benefits for individual group-mates.

Leadership is also likely to be strongly influenced by metabolic phenotypes. Within moving groups, individuals face a constant trade-off between leading the group towards their own desired target and potentially becoming fragmented from the majority of the group [114,115]. Leaders within groups are therefore likely to be those that are motivated towards a particular goal (e.g. to reach a food patch) or that are ‘socially indifferent’ (as per [114]). Both factors may be influenced by metabolic demand and locomotor capacity. Fish that have experienced short-term food deprivation, for example, are more likely to be at the front of moving shoals and thus directing movements of the group [79,80]. With longer term food deprivation, however, it appears that this may change as individuals begin to prioritize shoal cohesion, perhaps in response to reduced locomotor performance and a need for safety in numbers [23]. Fish with generally higher metabolic requirements (i.e. an elevated SMR) or increased capacity for threat detection or escape responses may also prioritize resource acquisition over sociability and lead movements of entire groups as they move towards their own preferred target destinations or modulate their speed and assertiveness [114].

These considerations may be especially important when groups face challenges such as food shortages, thermal shifts or hypoxic episodes in aquatic environments [6]. Groups of individuals with similar tolerances to these stressors should show greater cohesion up to their critical tolerance limits [17,116]. A notable exception, however, is that a group in which all individuals have a high SMR may experience more intense intragroup competition for food items when compared with a more heterogeneous group, again suggesting the non-random among-group assortment based on energy requirements or performance capacity may be stronger for lower performance individuals.

Group composition can also have impacts on social dynamics and must be considered when designing laboratory-based experiments on groups of animals. As familiarity is critical for a range of important processes [117–119], groups of animals should ideally not be haphazardly placed together shortly before testing. Furthermore, if groups in the wild show non-random assortment according to physiological phenotypes by active or passive means, then the composition of groups in the laboratory may not be representative of ecologically relevant group cohesion and leadership.

### (c) Resource allocation within individuals

Life-history theory dictates that animals vary their allocation of energy to processes including growth, activity and reproduction depending on factors such as age and environmental conditions (e.g. predator density and food availability) [120]. Although rarely considered in this context, an animal's social environment should also influence the proportional investment of these energy resources [121]. For example, a high-performance individual within a lower performance group may allocate energy away from costly somatic maintenance and performance capacity (e.g. skeletal muscle) to gonadal development. Individuals in appropriate group sizes with metabolically similar conspecifics may generally maximize net energy intake, with downstream effects on growth and reproductive investment that interact with factors traditionally considered by life-history theory. The extent to which these mechanisms mediate life-history traits via effects on physiology has not been investigated.

### (d) Group responses to environmental change

A greater understanding of the physiological composition of animal groups and the interplay between social dynamics and individual physiology will be key for predicting species' responses to environmental change. Within shoals, certain individuals tend to influence the directional movements of the entire group [122,123]. When tested individually, these leaders tend to be more bold and exploratory—two aspects of animal personality which, at least in some contexts, are positively linked to metabolic rate [5,123–125]. Interestingly, individuals with a higher metabolic rate may also be less tolerant of environmental stressors such as hypoxia, temperature increases and food deprivation [126,127]. As a result, environmental change could have a disproportionate effect on the overall behaviour of animal groups via increased physiological sensitivity of group leaders. Long-term shifts in factors such as temperature could change selective pressures on physiological tolerance to stressors and could even lead to genetic changes in populations for traits such as SMR, MMR or AS, all of which could also affect behaviour within schools. These changes could also shift the balance of mechanisms impacting physiological assortment patterns, potentially altering the trade-offs of varying physiological phenotypes within animal groups.

Storms and other extreme weather can cause animal groups to break up into smaller units [128,129]. Climate-associated increase in the frequency of extreme weather events [130] is likely to cause a corresponding increase in the rate at which animal groups split and reform with among-group mechanisms of assortment playing a key role. In fishes, evidence suggests that the stress of isolation due to an acute disturbance can lead to a rise in basic energetic needs [131].

### (e) Selective pressures and evolutionary trajectories

Active or passive assortment according to physiological traits could create a clustering of conspecifics with particular physiological phenotypes, with important implications for assortative mating within species and local adaptation. If environmental factors (e.g. food availability, temperature) covary with the distribution of phenotypes, then plasticity could further enhance physiological differences among groups or reveal phenotypic traits that would otherwise not normally be exposed to selection. Depending on the scale at which non-random assortment is influenced by physiological traits, scenarios could arise where different phenotypes are exposed to different selective pressures within different geographical regions within a species' range. Partial and diel migration may also be linked to metabolic phenotypes within populations (or to traits such as boldness which can be correlated with metabolic traits [5,132]), possibly generating large-scale non-random assortment and changes in gene flow and population demographics.

Within groups, the spatial location of individuals relative to group-mates will strongly affect the benefits they derive from group membership and the selection pressure that they experience. For example, the available evidence suggests that individuals near the front of moving fish schools may be more likely to experience predatory attacks, while those at the back tend to receive less or poorer quality food [133,134]. If different phenotypes consistently occupy particular spatial locations within groups, they may experience selection due to factors such as predation or resource availability. Interestingly, environmental conditions such as temperature or water flow rate could modulate the spatial positions occupied by particular phenotypes [17]. For example, fish with a high SMR may tend to be located near the front of schools at high temperatures to receive more food, but towards the back of the group at lower temperatures. This would result in context-dependent selection for or against particular physiological phenotypes.

## 5. Future directions

We have outlined numerous potential mechanisms by which physiological traits may influence non-random assortment both among- and within-fish shoals. There is still much work to be done to determine the extent to which these processes actually occur and their consequences. In the laboratory, recent advances in automated multi-agent tracking from video of animals in arenas [135] will provide an unprecedented opportunity to examine how physiological traits influence individual behaviour in groups, social networks, group decision-making, and group fission and fusion processes. Of particular interest will be understanding the costs and benefits of non-random assortment based on physiological traits and how the balance of the trade-offs involved in assortment may differ for individuals with varying phenotypes. In addition to empirical work in this area, a game theoretical approach is likely to be useful for generating predictions for how individuals of a given phenotype should opt to join groups of similar or dissimilar individuals.

An important but challenging area of research will be to measure physiological trait variation among and within shoals in the wild and to delineate the relative roles of active and passive processes in structuring the observed variation. New technologies in acoustic telemetry are facilitating the tracking of wild fish movements at spatial and temporal scales not previously possible [136]. In addition, the reduced incidence of signal collisions from acoustic transmitters permits an increased number of individuals that can be tracked within a given water body. Such data could be used to understand group behaviours of animals in the wild and its links with individual physiological traits [137]. Measures of metabolic traits can be measured on animals in the laboratory before being released for tracking, though there are also developing technologies for logging heart rate or using accelerometers to estimate energy expenditure in free swimming animals [138]. Experiments that also examine the effects of factors such as temperature and oxygen availability on group formation and assortment will be key in predicting animal responses to environmental change.
